# Association of TIP30 expression and prognosis of hepatocellular carcinoma in patients with HBV infection

**DOI:** 10.1002/cam4.728

**Published:** 2016-07-15

**Authors:** Xia Zhang, Lizhi Lv, Xuenong Ouyang, Shi'an Zhang, Jian Fang, Lirong Cai, Dongliang Li

**Affiliations:** ^1^ Department of HepatologyFuzhou General HospitalNanjing CommandFuzhou 350025China; ^2^ Department of Hepatobiliary SurgeryFuzhou General HospitalNanjing CommandFuzhou 350025China; ^3^ Department of OncologyFuzhou General HospitalNanjing CommandFuzhou 350025China

**Keywords:** HBV, hepatocellular carcinomas, Survival, TIP30, tumor suppressor

## Abstract

Altered expression of TIP30, a tumor suppressor, has been observed in many cancers. In this study, we have evaluated the expression of TIP30 in the tissues of 209 hepatocellular carcinomas (HCC) and their adjacent tissues by using a high‐density tissue microarray, and analyzed its correlation with the clinical pathological parameters of the patients. The results revealed negative or weak expression of TIP30 in 43.5% (91/209) of the HCC tissues, and in only 27% (56/209) of the adjacent tissues. The expression level of TIP30 in HCC was inversely correlated with serum alpha‐fetoprotein (AFP) levels, HBV infection, and tumor differentiation. Multivariate analysis for survival indicated that serum HBV infection was the most significant predictor of poor prognosis in HCC (*P *=* *0.0023), and TIP30 expression and tumor differentiation were also independent indicators in this respect (*P *=* *0.0364 and *P *=* *0.0397, respectively). Patients with medium or high expression levels of TIP30 (TIP30^++/+++^) had a better 5‐year overall survival rate than those with low/negative (TIP30^+/−^) expression (*P *<* *0.001). TIP30^+/−/^HBV^+^ patients had the worst 5‐year overall survival rate, whereas TIP30^++/+++^/HBV^−^ patients had the best. To further explore the correlation between TIP30 and HBV infection in HCC, HBV^+^ hepatoblastoma cell‐line HepG2 2.2.15 and HCC cell‐line Hep3B were used. Upon silencing of HBV, we observed an upregulation of TIP30 and decreased cell proliferation. In the in vivo studies, we found that the mice inoculated with HepG2 2.2.15 cells with HBV silencing had a prolonged tumor latency and a longer life span, as compared to the control mice inoculated with untreated control cells. In conclusion, the results suggest that downregulation of TIP30 may result from HBV infection, and subsequently promotes the progression of HCC.

## Introduction

Hepatocellular carcinoma (HCC) is one of the most common cancers worldwide. According to the World Health Organization (WHO), at least 550,000 people die each year from HCC 1. It has been the second leading cause of cancer‐related death in the world, and about 40% of these cases occur in China [2]. Chronic infection of the hepatitis B virus (HBV) has been linked epidemiologically to the development of HCC^3^, ^4^. It is estimated that in most Asia‐Pacific countries, chronic HBV infection accounts for 75–80% of all HCC cases 5.

Many studies have explored the mechanisms surrounding the determination of whether or not HBV infection is related to HCC. The HBV genome is a 3.2‐kb DNA molecule that contains four genes: C, S, P, and X. The HBV X gene encodes a multifunctional protein that plays a crucial role in hepatocarcinogenesis. The HBV X protein can activate many viral and cellular genes, modulate cellular signal transduction pathways, and regulate cell proliferation, apoptosis, and genetic stability 6, 7, 8, 9, 10, 11, 12. For example, the HBV X protein may promote the transformation of liver cells into cancer initiating cells through activating the Wnt/*β*‐catenin signaling pathway 8. It also interferes with nucleotide excision repair via both p53‐dependent and p53‐independent pathways 12. Furthermore, carboxyl‐terminal truncated X protein loses its inhibitory effects on cell proliferation and proapoptotic properties, and it may therefore enhance the protein's ability to transform normal cells into cancer cells 13, 14.

Tat‐interacting protein (TIP30), also called CC3, is a 30 kd protein first identified through a differential display analysis of mRNA from the highly metastatic human variant of small cell lung cancer (SCLC) cells as opposed to less metastatic classic SCLC cells 15. It has been reported that the expression level of TIP30 decreases in cases of lung cancer, HCC, colon cancer, breast cancer, and prostate cancer 16, 17, 18, 19. TIP30 acts as a transcription cofactor and regulates expressions of genes involved in apoptosis, cell growth, and tumor angiogenesis 20, 21, 22, 23. Tip30 mRNA expression may be downregulated by TIP30 promoter hypermethylation in HCC and has been associated with poor prognosis 24. In addition, TIP30 can inhibit HCC cell proliferation and induce apoptosis through modulating stabilization of p53 mRNA 20, 25. Further studies have revealed that downregulation of TIP30 has enhanced expression of osteopontin, as well as matrix metalloproteinase‐2 and the vascular endothelial growth factor 26. However, the precise mechanisms involved in TIP30 downregulation in HCC and its relationship with HBV still remain unclear.

In this study, we hypothesized that HBV infection might downregulate TIP30 expression and contribute to HCC development. We therefore examined TIP30 expression by immunohistochemistry in 209 HCC tissue samples using a high‐throughput tissue microarray. The correlations of TIP30 expression with TNM stage, tumor differentiation, serum HBV titer, and alpha‐fetoprotein (AFP) level were evaluated. The effects of TIP30 expression and serum HBV levels on the survival rates of HCC patients were also analyzed. Data obtained from the clinical analysis suggest that the downregulation of TIP30 may be partially due to HBV infection, and that it therefore promotes HCC progression. We further verified the clinical findings using analysis of both a cell culture system and a heterotopic nude mouse model.

## Materials and Methods

### Patients and tumor specimens

The surgically dissected tumor tissues and needle biopsy samples, as well as the corresponding clinical information were retrospectively retrieved for patients treated for HCC at Fuzhou General Hospital between 2005 and 2011. All patients had well‐documented clinical data and follow‐up information. None of the patients received preoperative chemotherapy or radiotherapy before operation or acupuncture. This study was approved by the Ethics Committee of the Fuzhou General Hospital, and all patients provided written informed consent prior to participation.

### Histology and Immunohistochemistry

Specimens were fixed in 10% buffered formalin and embedded in paraffin. The samples were then used to generate tissue microarray slides 27. Slides were stained with hematoxylin‐eosin for histological examination. TIP30 protein was assessed immunohistochemically. Tumor sections were deparaffinized in Histo‐Clear and hydrated in decreasing concentrations of ethanol. Endogenous peroxidase activity was quenched with 5% hydrogen peroxide in ethanol for 20 min. Nonspecific background staining was blocked with goat serum for 20 min. Then, the sections were incubated in the goat anti‐TIP 30 antibody (Santa Cruz Biotechnology, Santa Cruz, CA), at a dilution of 1:100 overnight at 4°C. The primary antibody was replaced with nonimmune goat serum in a negative control. After washing, a corresponding biotinylated secondary antibody (Dako, Ely, Cambridgeshire, UK) was then applied at a dilution of 1:500 for 2 h at room temperature. A horseradish eroxidase system was used as a secondary detection system with diaminobenzidine (DAB). Negative controls were performed simultaneously by omitting the primary antibody for each specimen. Assessment of the immunohistochemical reaction was performed according to the number of positively stained cells. Each specimen was assigned to one of the four following categories: (1) negative (−), when fewer than 10% of the cells were stained; (2) weak (+), when stained cells accounted for 10–25% of the total number of cells; (3) moderate (++), when stained cells accounted for 50–75% of the total number of cells; and (4) strong (+++), when more than 75% of the cells were positively stained. The results were evaluated separately by two of the authors (XZ and LW), without any previous knowledge of the clinical information of each patient. Clinical data were analyzed according to gender, age, TNM stage, serum HBV infection, AFP level, tumor differentiation, and TIP30 expression.

### Cell culture and transfection

The hepatoblastoma cell‐line cell HepG2 2.2.15 (Aiyan Bio‐Science Company, Shanghai, China) and the HCC cell‐line Hep3B (Oncology Center of Shanghai Hospital, Shanghai, China), which are both HBV positive 28, 29, 30, were maintained in Dulbecco's Modified Eagle's medium (DMEM), containing 10% FBS (Sigma), and cultured at 37°C in an atmosphere containing 95% air and 5% CO_2_ and passaged twice a week. Both cell lines were stably transfected with a head‐to‐tail dimer of HBV DNA.

### Transfection of siRNA

The synthesizing of siRNAs was conducted by Sangon Co. Ltd (Shanghai, China). In addition, dsRNAs of HBV polyadenylation (PA) siRNA targeted to the polyadenylation signal of the HBV genome were used. A random sequence of dsRNA with the same overall length and the same length of overhanging ends served as the control. The sequence of the HBVPA siRNA was as follows: ACCCUUAUAAAGAAUUUGGdAdG; dGdCUGGGAAUAUUUCUUAAACC.

To assess the effects of siRNA on HBV viral gene expression, HepG2 2.2.15 cells (1.0 × 10^5 ^cells/mL) were incubated for 24 h in DMEM with 10% fetal bovine serum in 3.5 cm dishes. Oligofectamine of 3 *μ*L (Invitrogen, Carlsbad, CA) was mixed with 27 *μ*L of opti‐MEM medium (Invitrogen, Carlsbad, CA) and incubated for 10 min at 25°C. In another tube, 80 *μ*mol of siRNA was mixed with 102 *μ*L of opti‐MEM medium. These two solutions were gently mixed, and incubated for 20 min at 25°C. Then, 64 *μ*L of opti‐MEM medium was added to obtain a final volume of 200 *μ*L (0.4 pmol/L siRNA). Cells were washed with sterile phosphate‐buffered saline (PBS) twice, and incubated with siRNA–oligofectamine complex in DMEM without fetal bovine serum for 18 h at 37°C. Then, DMEM with Fetal bovine serum was added to make up a final concentration of 10% fetal bovine serum.

In other experiments, 72 h after first transfection, the cells were replated at a density of 1 × 10^5^ cells/mL in 3.5 cm dishes, and a second round of transfection was performed. Cell layers and medium were collected at 1, 3, 5, and 7 days after initiation of transfection, and were stored frozen at −80°C for further analysis. Control dishes were treated in an identical manner with siRNA alone, oligofectamine alone, random dsRNA–oligofectamine complex, or opti‐MEM alone as an untreated control. The inhibitory effects were analyzed in comparison with untreated control.

To assess the effect of siRNA on HBV protein expression levels, the hepatitis B surface antigen (HBsAg) concentrations in the culture medium were determined using an Auszyme Monoclonal Diagnostic Kit (Abbott Diagnostics, North Chicago, IL) according to the manufacturer's instructions. Assays of 200 *μ*L of DMEM were normalized to the numbers of cells per dish. Assays were performed in triplicate, expressed as means ± SD and relative to the untreated control (as 100%).

### Cell proliferation and viability assay

MTT [3‐(4, 5‐dimethylthiazol‐2‐yl)‐ 2,5‐ diphenyltetrazolium bromide] assay (Sigma, St. Louis, MO) was used to examine cell proliferation. Briefly, at different times (1–7 days) posttransfection, MTT (0.25 mg/mL) was added to each well and the plates were incubated for 4 h at 37°C and 5% CO^2^. After removing the MTT solution, 180‐*μ*L dimethyl sulfoxide (DMSO) was added and the plates were incubated for 15 min at 37°C to dissolve the formazan crystals. The absorbance values were measured at 570 nm using an automated ELISA reader (Bio‐Rad, Hercules, CA). Alternatively, cell viability was evaluated with the trypan blue staining assay.

### Western blot analysis

Total cell lysate was prepared in 1 × SDS buffer. Proteins were separated by 12% SDS polyacrylamide gel electrophoresis and transferred onto polyvinylidene difluoride membranes. Membranes were then blotted with individual antibodies. Antigen–antibody complexes were visualized with the enhanced chemiluminescence reagent Supersingal (Pierce Biotechnology, ockford, IL). The antibodies used were anti‐TIP30 (R&D Systems, Minneapolis, MN) and anti‐beta‐catenin (KangChen Biotech, Shanghai, China).

### Tumorigenicity assay

Nude female Balb/c mice of 4–6 weeks old were purchased from the Shanghai Experimental Animal Center of the Chinese Academy of Sciences (Shanghai, China). Animals were placed in a pathogen‐free environment and allowed to acclimate for a week before being used in the study. All procedures were performed according to institutional guidelines and conformed to the National Institutes of Health guidelines on the ethical use of animals.

Twenty‐four hours after adenovirus infection, HepG2 2.2.15 cells were trypsinized and resuspended in PBS. About 1 × 10^7^ cells in a 200‐*μ*L volume were injected subcutaneously per mouse (*n* = 6 for each group). Tumor size was determined weekly as [length (mm) × width (mm)^2^]/2. The survival of each animal was monitored and recorded.

### Statistical analysis

The data were analyzed using a *t* ^*2*^‐test to determine the differences between the groups using StatView statistical software (version 5.0.1; SAS Institute, Cary, NC, USA). To determine the relative survival of patients, the Cox's proportional hazards regression model was used, and survival curves after surgery were obtained using the Kaplan–Meier method. Statistical comparison of survival was performed using the log‐rank test. The *P* values <0.05 were considered statistically significant.

## Results

### Correlation of TIP30 expression in HCC with clinical pathological factors

There were 141 male and 68 female patients. The mean age was 51.44 ± 8.52 years. The follow‐up period of the patients ranged from 3 to 36 months. TIP30 expression was investigated in the 209 tissue samples and no correlation of TIP30 expression was found with respect to the patients’ ages, genders, and tumor histological classifications. However, there were significant inverse correlations between TIP30 expression and serum AFP level, tumor differentiation, and serum HBV infection (Table 1).

**Table 1:** The correlation between TIP30 expression and clinicopathological features of HCCs

Variable	TIP30 + /− (*n* = 91)	TIP30 + +/+++(*n* = 118)	*P* value
Gender			
Male	49	62	
Female	42	56	0.851
Age (years)	87	122	
≥60	37	49	
<60	54	69	0.900
TNM stage			
I–II	34	44	
III–IV	57	74	0.991
Serum AFP			
450 ng/mL	42	29	
>450 ng/mL	49	89	0.001
Serum HBV			
Negative	12	36	
Positive	79	82	0.001
Differentiation			
Well	8	41	0.000
Moderate and Poorly differentiated	83	77	

We also explored the survival rates of the 209 HCC patients using univariate analysis. Univariate analysis revealed that age and gender had no prognostic significance for overall survival (OS). In this HCC population, serum HBV and TIP30 expression were independent prognostic factors for OS (*P *<* *0.0001 and *P *<* *0.001, respectively). The Multivariate Cox modeling confirmed TIP30 expression as an independent predictor of survival (HR: 11.71, 95% CI: 6.63–18.76, *P *<* *0.001, Table 2). In addition, the AFP level and tumor differentiation were also significant predictors (*P *<* *0.001, and *P *<* *0.01, respectively).

**Table 2:** Univariate and multivariate survival analysis for the HCC patients (*n* = 209)

Variable	Univariate analysis*P*‐value	Multivariate analysis (Cox Regression)HR CI (95% low–high limits)		*P*‐value
TIP30 expression	<0.001	11.71	6.63–18.76	<0.001
Tumor size (>35 mm)	<0.001			
Vascular invasion	<0.001			
Albumin levels (<3.5 gr/L)	0.03			
AFP levels (>450 ng/mL)	<0.001			
TNM stage	<0.001	2.74	2.63–3.15	0.04
Serum HBV	<0.001			

**Figure 1:**
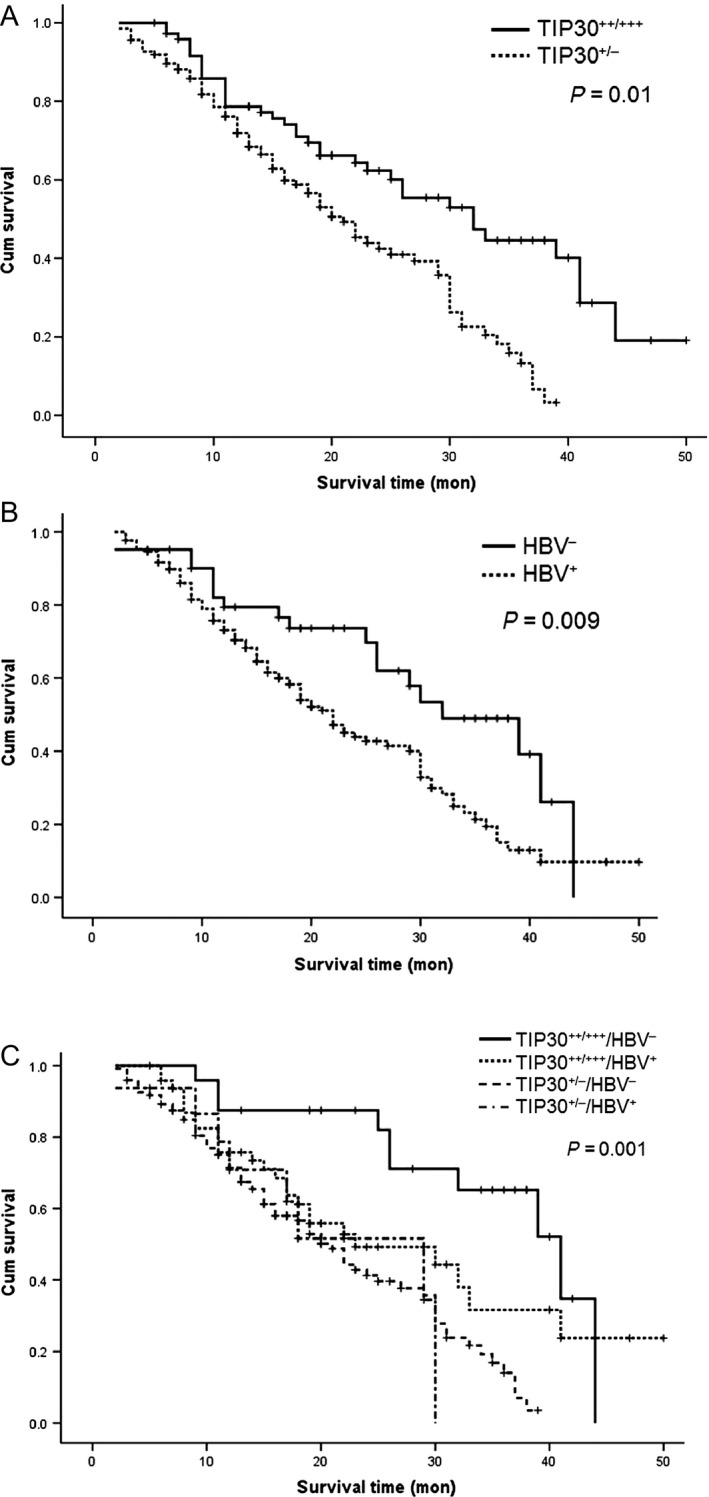
Kaplan–Meier estimates of survival in patients with hepatocellular carcinoma. (A) Patients with TIP30 weak/negative (TIP30^+/−^) expression (*n* = 91) had a worse 5‐year overall survival rate (OS) than those with medium or high TIP30 expression (TIP30^++/+++^) (*n* = 118) (*P *=* *0.01). Median OS was 21.8 months versus 29.2 months; (B) Patients without HBV infection (HBV^−^, *n* = 48) had a better 5‐year OS than those with HBV infection (HBV^+^, *n* = 161) (*P *=* *0.009). Median OS was 36.3 months versus 21.4 months; (C) Patients in the TIP30^++/+++^/HBV^−^ group had the best 5‐year OS than those in the TIP30^++/+++^/HBV^+^, TIP30^+/−^/HBV^−^, and TIP30^+/−^/HBV^+^ groups (*P *=* *0.001). Median OS was 40.9 months, 30.7 months, 29.0 months, and 21.6 months, respectively.

Further analysis showed that patients with TIP30 weak/negative (TIP30^+/−^) expression had a worse 5‐year OS than those with medium or high expression of TIP30 (TIP30^++/+++^) (Fig. 1A). Patients with HBV infection had a worse OS than those without HBV infection (Fig. 1B). Layer analysis revealed that the patients in the TIP30^+/−^/HBV^+^ subgroup had the worst 5‐year OS among all subgroups, whereas the patients in the TIP30^++/+++^/HBV^−^ subgroup had the best OS. (Fig. 1C)

### Downregulation of HBsAg expression increased TIP30 expression in HCC cells

From the clinical studies, we found that in patients with HBV infection, the expression levels of TIP30 were decreased in the tumor tissue samples. A likely hypothesis is that HBV may downregulate TIP30 expression in order to promote HCC progression. To test this hypothesis, we performed the following experiments with two HBV‐positive lines: the hepatoblastoma cell‐line HepG2 2.2.15 and the HCC cell‐line Hep3B.

**Figure 2:**
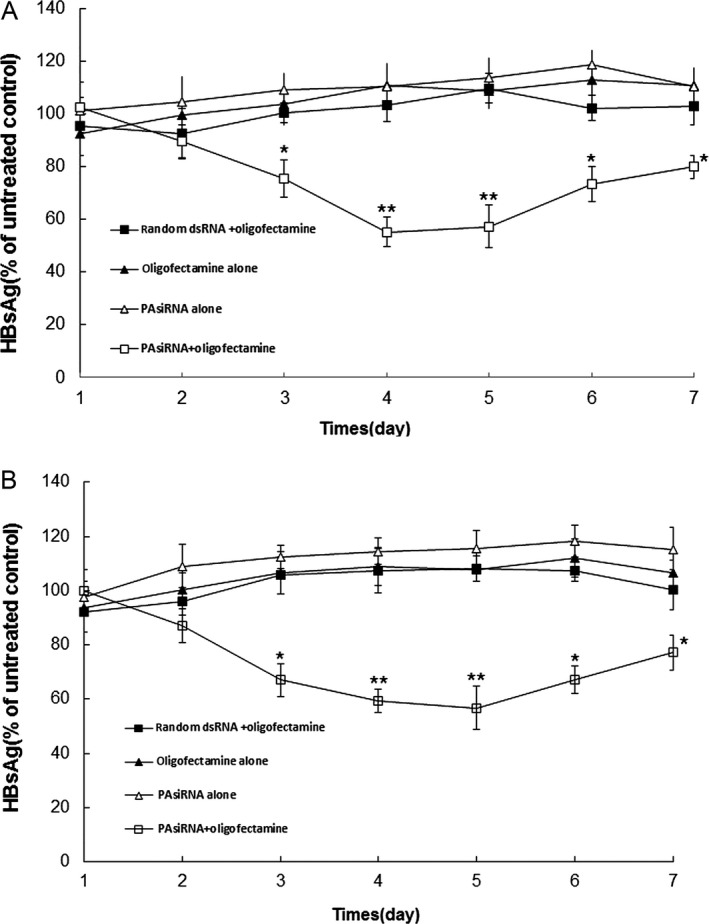
The effect of HBV siRNAs on HBV gene expression. HBsAg concentrations in the culture media of HepG2 2.2.15 (A) and Hep3B cells (B) were measured after transfection with HBV PA siRNA–oligofectamine and controls. *P <* *0.05, **P <* *0.01 versus random dsRNA + oligofectamine.

To evaluate the effects of HBV siRNAs on HBV gene expression, HBsAg concentrations in the culture media of both treated and control cells were measured after transfection using a standard HBsAg kit. This process revealed that the inhibition rates of HepG2 2.2.15 treated with the HBVPA siRNA–oligofectamine complex were 35%, 47%, 47%, and 23%, respectively, at 3, 4, 5, and 6 days after transfection, as compared to the results of untreated control group (Fig. 2A). For Hep3B cells treated with HBVPA siRNA–oligofectamine complex, the inhibition rates were approximately 31%, 40%, 50%, and 27%, respectively, at 3, 4, 5, and 6 days after transfection, as compared to the results of the untreated control (Fig. 2B). For each cell line and nearly all points in time, the inhibition rates by transfection of HBVPA siRNA–oligofectamine were all significantly higher than those by transfection of HBVPA siRNA alone or oligofectamine alone or random dsRNA–oligofectamine complex.

**Figure 3:**
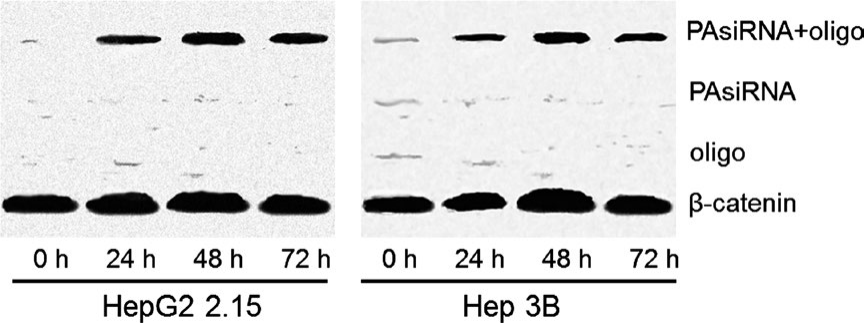
The TIP30 expression of HepG2 2.2.15 and Hep3B cells before and after HBV silencing with siRNA. Western blot results showed that TIP30 expression was detected 24 h after HBV silencing with siRNA, and peaked at 48 h.

We next examined the TIP30 expression levels before and after HBV silencing with siRNA. The results showed that after HBV was decreased by siRNA, the expression levels of TIP30 were significantly enhanced in both HepG2 2.2.15 and Hep3B cells in a time‐dependent manner (Fig. 3), which corresponded to the results of time‐dependent suppression of HBV by RNA silencing.

### HBV siRNA interference suppressed the proliferation of HepG2 2.2.15 and Hep3B cells

**Figure 4:**
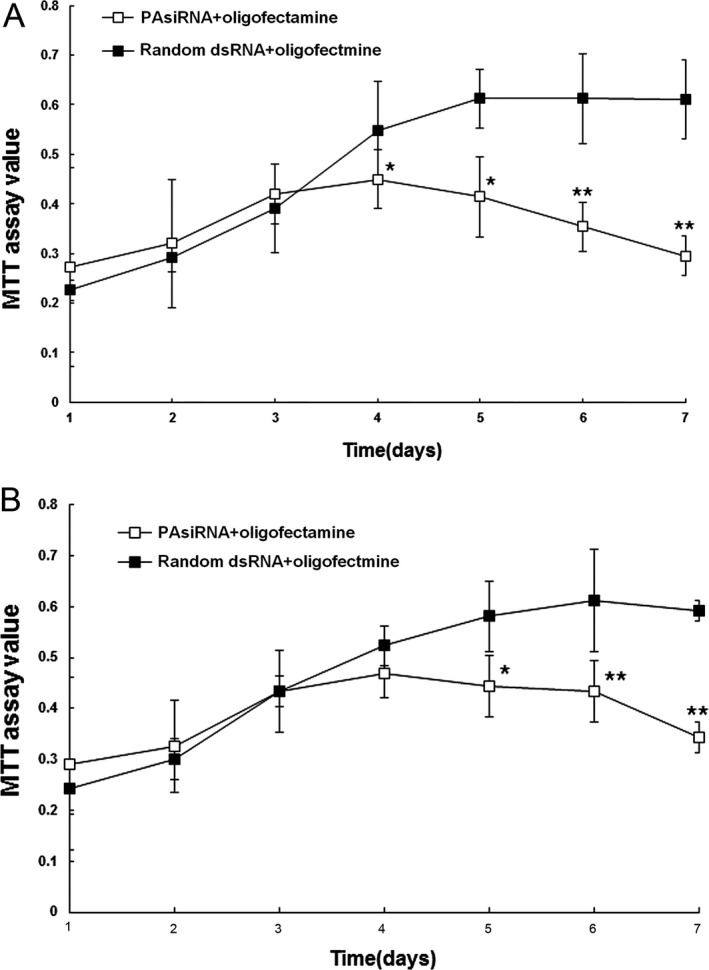
Cell growth assay of HepG2 2.2.15 (A) and Hep3B cells (B) transfected with HBVPA siRNA–oligofectamine. After HBV was interfered, both cells grew at a much slower rate than the cells transfected with random dsRNA–oligofectamine complex. *P <* *0.05,**P <* *0.01 versus random dsRNA +oligofectamine.

The proliferation of HepG2 2.2.15 and Hep3B cells transfected with HBVPA siRNA–oligofectamine (named as HepG2 2.2.15‐TIP30^+^ and Hep3B‐TIP30^+^, respectively) were then examined. As shown in Figure 4A, HepG2 2.2.15‐TIP30^+^ cells grew at a much slower pace than the HepG2 2.2.15 cells transfected with random dsRNA–oligofectamine complex. Similar results were observed for Hep3B cells (Fig. 4B). At day 5 post‐transfection with HBVPA siRNA–oligofectamine, the numbers of HepG2 2.2.15‐TIP30^+^ and Hep3B‐TIP30^+^ cells were about 30% and 24% lower, respectively, as compared to the numbers of control cells. At day 7, the numbers of HepG2 2.2.15‐TIP30^+^ and Hep3B‐TIP30^+^ cells were about 50% and 39% lower, respectively, as compared to the numbers of control cells.

### HBV siRNA interference suppressed the growth of HCC cells in nude mice

**Figure 5:**
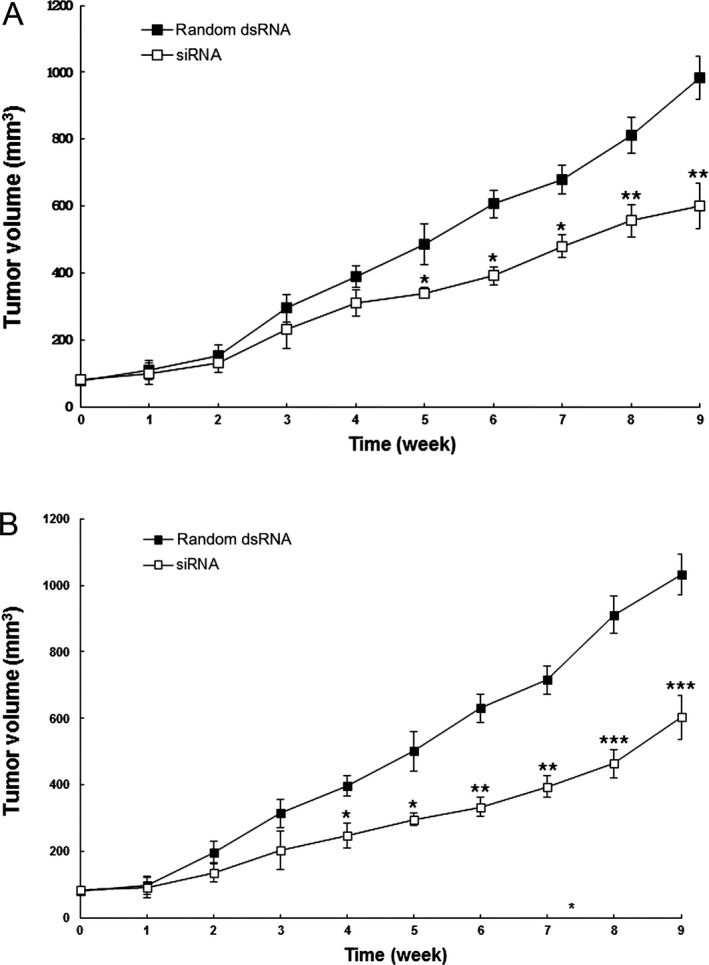
Growth assay of HepG2 2.2.15 and Hep3B cells transfected with HBVPA siRNA–oligofectamine in nude mice. HepG2 2.2.15 (A) and Hep3B (B) cells transfected with HBVPA siRNA–oligofectamine grew significantly slower in nude mice as compared to the control cells. *P <* *0.05, **P <* *0.01, ***P <* *0.001 versus untreated cells.

**Figure 6:**
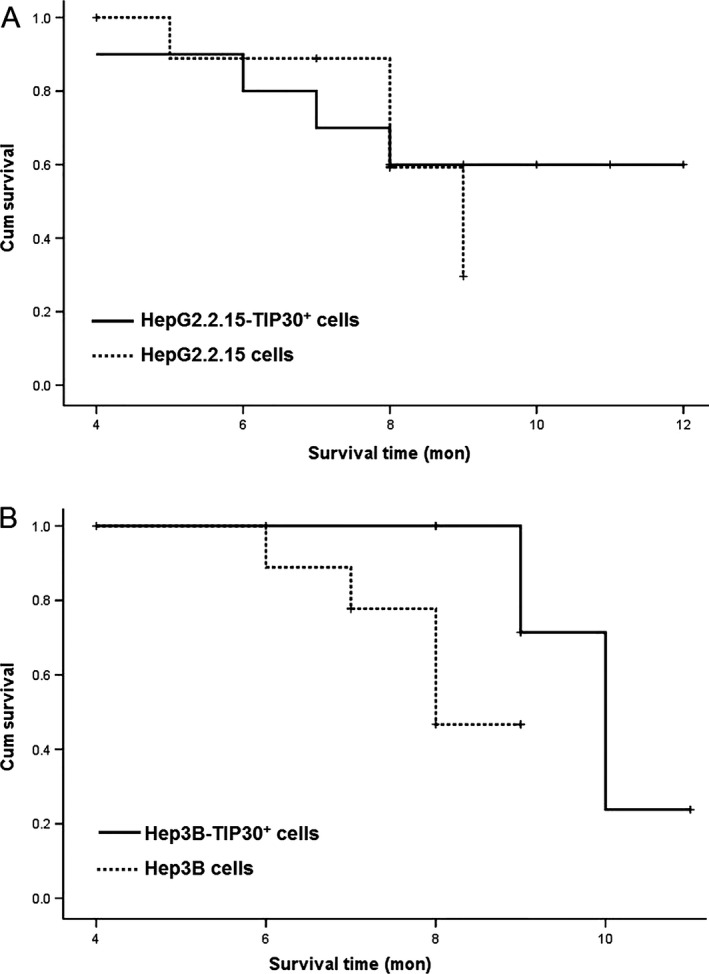
Survival curve of mice. (A) Mice were injected s.c. with 2 × 10^6^ HBV siRNA‐transfected HepG2 2.2.15 cells or with wild‐type HepG2 2.2.15 cells; (B) Mice were injected s.c. with 2 × 10^6^ HBV siRNA‐transfected Hep3B cells or with wild‐type Hep3B cells. Mice injected with HBV siRNA‐transfected cells had a longer life span than those injected with wild‐type cells. Data were evaluated using a Kaplan–Meier survival curve and log‐rank test.

To determine the suppressive tumor activity of HBV silencing in vivo, we then studied the growth of HepG2 2.2.15‐TIP30^+^ and Hep3B‐TIP30^+^ cells in nude mice. After inoculation with the same cell numbers, we found that it took a longer time for HepG2 2.2.15‐TIP30^+^ and Hep3B‐TIP30^+^ cells to develop tumors than it did in the control cells (Fig. 5). The HBV interfered HepG2 2.2.15 and Hep3B cells, which had an upregulated TIP30, and both grew significantly slower as compared to the control cells in nude mice (Fig. 5B). Mice implanted s.c. with HepG2‐TIP30^+^ and Hep3B‐TIP30^+^ cells had a longer life span than those implanted with control cells (Fig. 6). These data suggest that HCC cells interfered with HBV siRNA‐enhanced TIP30 expression, and grew much slower in vivo, which confirms the results of our cell culture studies in vitro.

## Discussion

HCC, which occurs at an increasing frequency in developing countries, especially in China, is an aggressive cancer with high mortality. Many epidemiological studies suggest that HBV infection is the main risk factor for HCC development. Data from Taiwan which revealed that childhood vaccination programs against HBV dramatically decreased the incidence rates of liver cancer further confirmed this conclusion 31. Consequently, the World Health Organization includes HBV in ‘Group 1’ human carcinogens, thus classifying it among the most important oncogenic agents after tobacco smoking. In accordance with the previous studies, we found that HBV infection was an independent prognostic factor for OS of HCC patients.

HBV belongs to the Hepadnaviridae family and is one of the smallest viruses in nature. Several HBV proteins have been demonstrated to have prooncogenic activities. HBV may integrate into the genome of the infected host hepatocytes 32, 33. HBV seems to behave like an insertional, nonselective mutagenic agent that may induce host genome instability 34. In addition, integrated HBV DNA might produce mutated viral proteins such as truncated X proteins or preS/S proteins which may activate signaling pathways involved in hepatocarcinogenesis 35, 36, 37. Finally, insertion of HBV DNA into cellular genomic regulatory regions or coding regions may alter gene expression or the structure and function of the produced cellular proteins involved in tumorigenesis 38, 39, 40, 41, 42. This study showed that serum HBV were independent prognostic factors for OS, and patients with HBV infection had a worse OS than those without HBV infection. Furthermore, the HBV siRNA interference suppressed the growth of HepG2 2.2.15 and Hep3B cells in vitro and in vivo. HBV‐related HCC development underlies the complex and multifactorial pathogenetic mechanisms of HCC.

Association of TIP30 and carcinogenesis is well documented 16, 17, 22, 43. Thus, it is not surprising to find that downregulation of TIP30 was associated with the worse OS in HCC patients. In this study, we found no TIP30 expression in 91 out of 209 cases of HCC. Downregulation of TIP30 in the HCC cases was not correlated with patients’ age, gender, and TNM stages; however, it was inversely correlated with serum HBV infection, serum AFP, and tumor differentiation. The patients with medium or high TIP30 expression (TIP30^++/+++^) had a longer life span than those with weak/negative TIP30 (TIP30^+/−^) expression. The median survival period of patients with TIP30^++/+++^ expression was 12.9 months, and that of patients with TIP30^+/−^ expression was only 7.5 months. Layer analysis revealed that patients with TIP30^+/−^/HBV^+^ expression had the worst 5‐year overall survival rate among all groups, whereas the patients with TIP30^++/+++^/HBV^−^ had a best overall survival rate. By RNA interference, we confirmed our hypothesis that HCC might downregulate the expression of TIP30, although the underlying mechanisms are not clear. The possible mechanisms may include that TIP30 can suppress metastasis 18, 19, 26, 44, apoptosis 20, 45, oncogene activity 46, and cancer cell proliferation 21. TIP30 also inhibits tumor metastasis through regulating on angiogenesis and extracellular matrix 26, 47, 48, 49. In addition, Lu et al. showed that the inactivation of Tip30 gene expression by CpG island DNA hypermethylation may play a role in the hepatocarcinogenesis 24. Moreover, recent evidences indicate that TIP30 may be involved in the regulation of epithelial‐mesenchymal transition (EMT). Downregulation of TIP30 leads to EMT and induces tumor‐initiating cells, thus promoting the tumor metastasis in HCC 50.

Zhao et al., previously, found that there was no significant correlation between TIP30 expression and HBV infection 26. However, in this study, through analysis of the clinical data of HCC patients, we demonstrated a reverse relation between HBV infection and TIP30 expression in HCC. Furthermore, the cellular experiments showed that the TIP30 expression was significantly increased after HBV silencing with siRNA in a time‐dependent manner. Therefore, HBV may promote HCC progression by downregulating TIP30 expression. The results have shed new light on the development of HBV‐related HCC. Further studies are needed to elucidate how HBV infection actually downregulate TIP30 expression.

In conclusion, our study demonstrates that downregulation of TIP30 in HCC may result from HBV infection, and is an independent poor prognostic factor for the OS of HCC patients. The data have shed new light on the mechanisms involved in HBV‐related hepatocarcinoma, and will provide new strategies to the prevention of HBV‐related hepatocarcinogenesis and its progression.

## Conflict of Interest

The authors declare no conflict of interest.
